# Rare disease mimicking multisystem inflammatory syndrome in children

**DOI:** 10.1186/s12887-025-06451-5

**Published:** 2025-12-15

**Authors:** Asuman Akar

**Affiliations:** Faculty of Medicine DIYARBAKIR, Pediatric Infectious Disease, Dicle University, DIYARBAKIR, Diyarbakir, Turkey

**Keywords:** Multisystem inflammatory syndrome, Visceral leishmaniasis, SARS-CoV-2, Differential diagnosis, Immune dysregulation

## Abstract

Multisystem inflammatory syndrome (MIS-C) is a rare but serious condition associated with SARS-CoV-2, the virus that causes COVID-19. We report a 16-year-old female who presented with seven days of fever, abdominal pain, headache, and fatigue. Physical examination revealed meningeal irritation. Laboratory findings showed pancytopenia, elevated inflammatory markers, and positive SARS-CoV-2 IgG with negative PCR. The patient had no history of COVID-19 vaccination or previous confirmed infection. Initially evaluated as MIS-C due to fever, multisystem involvement, and antibody positivity, she was subsequently diagnosed with visceral leishmaniasis after bone marrow aspiration revealed Leishmania amastigotes. This case highlights the diagnostic overlap between MIS-C and endemic infections such as leishmaniasis and underlines the importance of considering infectious etiologies in hyperinflammatory presentations, especially in endemic areas.

**Supplementary Information:**

The online version contains supplementary material available at 10.1186/s12887-025-06451-5.

## Introduction

In December 2019, a new coronavirus, Severe Acute Respiratory Syndrome Coronavirus-2 (SARS-CoV-2), was detected in Wuhan, China [1]. The World Health Organization (WHO) declared the outbreak a pandemic on March 11, 2020 [2]. Shortly afterward, clinicians reported an increasing number of children presenting with a multisystem inflammatory disease resembling Kawasaki disease and toxic shock syndrome [3]. Between May 22 and May 29, 2020, working groups from the Centers for Disease Control and Prevention (CDC), the WHO, and the Royal College of Paediatrics and Child Health (RCPCH) described this condition as Multisystem Inflammatory Syndrome in Children (MIS-C) associated with COVID-19 [4–6]. The syndrome typically develops four to six weeks after SARS-CoV-2 infection and is considered a result of immune dysregulation rather than acute viral disease [7]. Because MIS-C shares features with infectious and autoimmune conditions, its diagnosis can be challenging in endemic regions. This report describes a pediatric patient initially managed as MIS-C who was later diagnosed with visceral leishmaniasis, emphasizing the importance of comprehensive differential diagnosis.

## Case presentation

**Fig. 1:**
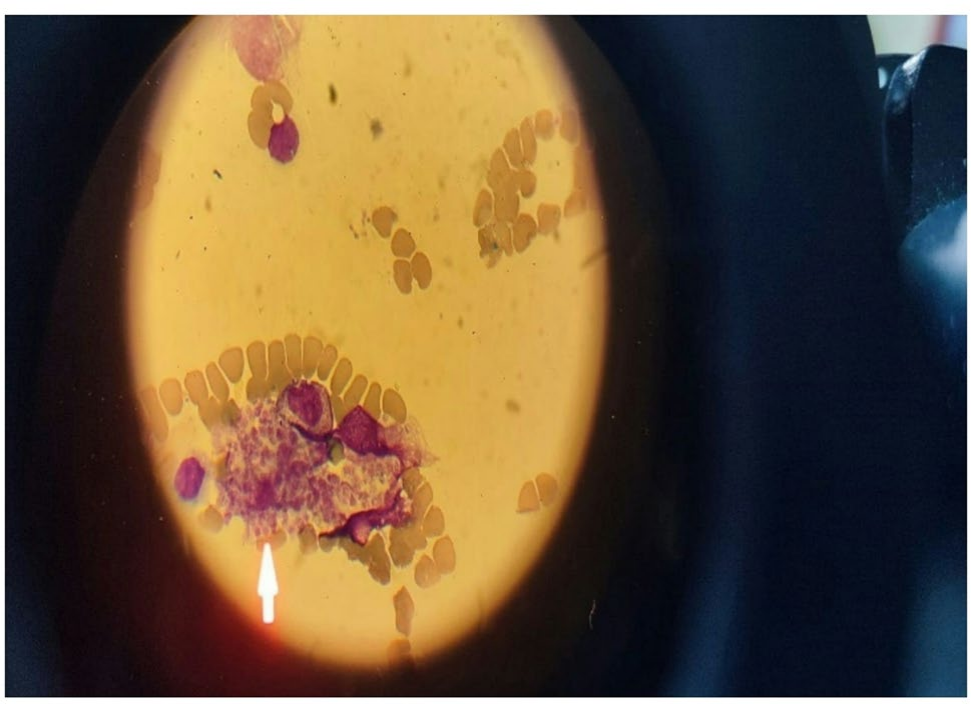
Bone marrow aspiration revealed a phagocytic cell containing amastigotes (Wright and Giemsta stain, ×1000)

A 16-year-4-month-old female with no previous medical history was admitted to the Emergency Room with seven days of fever (40 °C), abdominal pain, headache, and fatigue. She had never received a COVID-19 vaccine and had no known history of previous SARS-CoV-2 infection or exposure. On examination, her general condition was poor, with a frail appearance, neck stiffness, and herpes labialis. Other system findings were unremarkable. Vital signs: respiratory rate 20/min, blood pressure 100/60 mmHg, oxygen saturation 99%, GCS 15. Laboratory results were as follows: WBC 1530/mm³, neutrophils 610/mm³, lymphocytes 230/mm³, hemoglobin 10.2 g/dL, platelets 27,000/mm³, D-dimer 12,790 ng/mL, CRP 59.5 mg/L, procalcitonin 0.68 ng/mL, ESR 53 mm/h, ferritin 3176 µg/L, triglycerides 268 mg/dL, fibrinogen 100 mg/dL, AST 321 U/L, ALT 344 U/L, LDH 4264 U/L. Nasopharyngeal COVID-19 PCR was negative, and no pathogen was detected in the respiratory virus panel. Rapid antibody testing showed SARS-CoV-2 IgM negative and IgG positive, indicating previous exposure. Because of fever, multisystem involvement, and antibody positivity, the initial diagnosis was MIS-C. Lumbar puncture was performed due to signs of meningeal irritation: CSF glucose 54 mg/dL (serum glucose 96 mg/dL), protein 21 mg/dL, and no cells were observed on direct examination. No growth was found in cultures. Due to cytopenia and suspicion of secondary HLH-MIS-C, IVIG (2 g/kg) was administered. Broad infectious testing—including HIV, hepatitis, Epstein-Barr virus, cytomegalovirus, toxoplasma, parvovirus B19, brucella, and adenovirus serologies—was negative. Evaluation for possible immunodeficiency (serum immunoglobulin levels and lymphocyte subsets) was normal. Abdominal ultrasonography revealed no hepatosplenomegaly, and echocardiography was normal. On the third hospital day, bone marrow aspiration was performed due to persistent pancytopenia and poor IVIG response, revealing phagocytic cells containing Leishmania amastigotes (Fig. 1). Liposomal amphotericin B (3 mg/kg/day) was initiated; by day 3 of treatment, the fever subsided, and blood counts improved. Bone marrow aspiration amastigote was seen.

Patient classified as visceral leishmania. The patient was diagnosed with visceral leishmaniasis and initiated on liposomal amphotericin B at 3 mg/kg/day. Clinical improvement was evident by day 3 of therapy, with resolution of fever and amelioration of pancytopenia. A 7-day treatment course was completed.

## Discussion

There is a wide clinical spectrum in patients with MIS-C [8]. The diagnosis is made when fever, elevated inflammatory markers, and multisystem involvement are present in patients under 19 years of age with laboratory evidence of SARS-CoV-2 infection or exposure, after excluding alternative diagnoses [9]. In this case, the patient met MIS-C criteria but was ultimately diagnosed with visceral leishmaniasis, demonstrating the diagnostic overlap between hyperinflammatory syndromes and endemic infections. Visceral leishmaniasis is a zoonosis caused by *Leishmania donovani* and *L. infantum*, transmitted to humans by Phlebotomus flies [10]. It remains one of the most neglected tropical diseases, with an estimated 0.2–0.4 million cases annually [11, 12]. Typical findings include fever, pancytopenia, hepatosplenomegaly, and elevated inflammatory markers. Laboratory abnormalities include leukopenia, neutropenia, lymphocytosis, and elevated transaminases. Hemophagocytic lymphohistiocytosis has been reported in approximately 2% of cases, occasionally accompanied by mild neurological symptoms [13, 14]. In studies from Sudan, up to 4% of visceral leishmaniasis patients lacked splenomegaly, emphasizing the need to consider this diagnosis in cases of prolonged fever even without organomegaly [15]. The differential diagnosis includes miliary tuberculosis, brucellosis, typhoid, salmonellosis, infective endocarditis, infectious mononucleosis, collagen tissue diseases, and lymphoma, often leading to diagnostic delays [16]. Definitive diagnosis is established by identifying Leishmania amastigotes in bone marrow, spleen, liver, or lymph node aspirates [17]. Serological methods such as the direct agglutination test (DAT), immunofluorescence assay (IFA), and recombinant K39 antigen tests show variable sensitivity [18]. Molecular diagnostic tools, including direct-on-blood PCR and nucleic acid lateral flow immunoassays, are promising for future use [19]. Treatment options include antimonial compounds and miltefosine, though liposomal amphotericin B remains preferred due to lower toxicity and resistance risk [20]. In this case, Leishmania infection caused immune dysregulation characterized by macrophage activation, elevated cytokines, and impaired Th1 responses, clinically resembling MIS-C and secondary HLH. Although SARS-CoV-2 IgG indicated previous exposure, there was no evidence of active infection or immune deficiency, emphasizing that COVID-19 serology alone cannot confirm MIS-C in endemic regions.

## Conclusion

In regions where zoonotic infections such as leishmaniasis are endemic, visceral leishmaniasis should be considered in febrile children with MIS-C–like hyperinflammatory presentations, even without organomegaly. Early diagnostic evaluation, including bone marrow examination, is essential for timely management and favorable outcomes.

## Supplementary Information

Supplementary Material 1.

## Data Availability

The datasets generated during the current study are available from the corresponding author on reasonable request.

## Abrevations

SARS-CoV-2: Severe Acute Respiratory Syndrome Coronavirus-2
COVID-19: SARS-CoV-2-induced coronavirus disease
MIS-C: Multisystem İnflammatory Syndrome İn Children
KD: Kawasaki Disease
CDC: United Stataes Centers for Disease Control and Prevention
WHO: World Health organization
RCPCH: Royal College of Paediatrics and Child Health
CRP: C-reactive protein
IVIG: Intravenous İmmunglobuline
PCR: Polymerase Chain Reaction
HLH: hemophagocytic lymphohistiocytosis
DAT: Direct agglutination test
IFA: immunofluorescence assay
IFAT: immunofluorescence antibody test
HIV: Human Immunodeficiency Virus
