# Supplementary figures and images for: Rare disease mimicking multisystem inflammatory syndrome in children
Source: BMC Pediatr. 2025 Dec 15;26:56. doi: 10.1186/s12887-025-06451-5 (PMC12829272; doi:10.1186/s12887-025-06451-5)

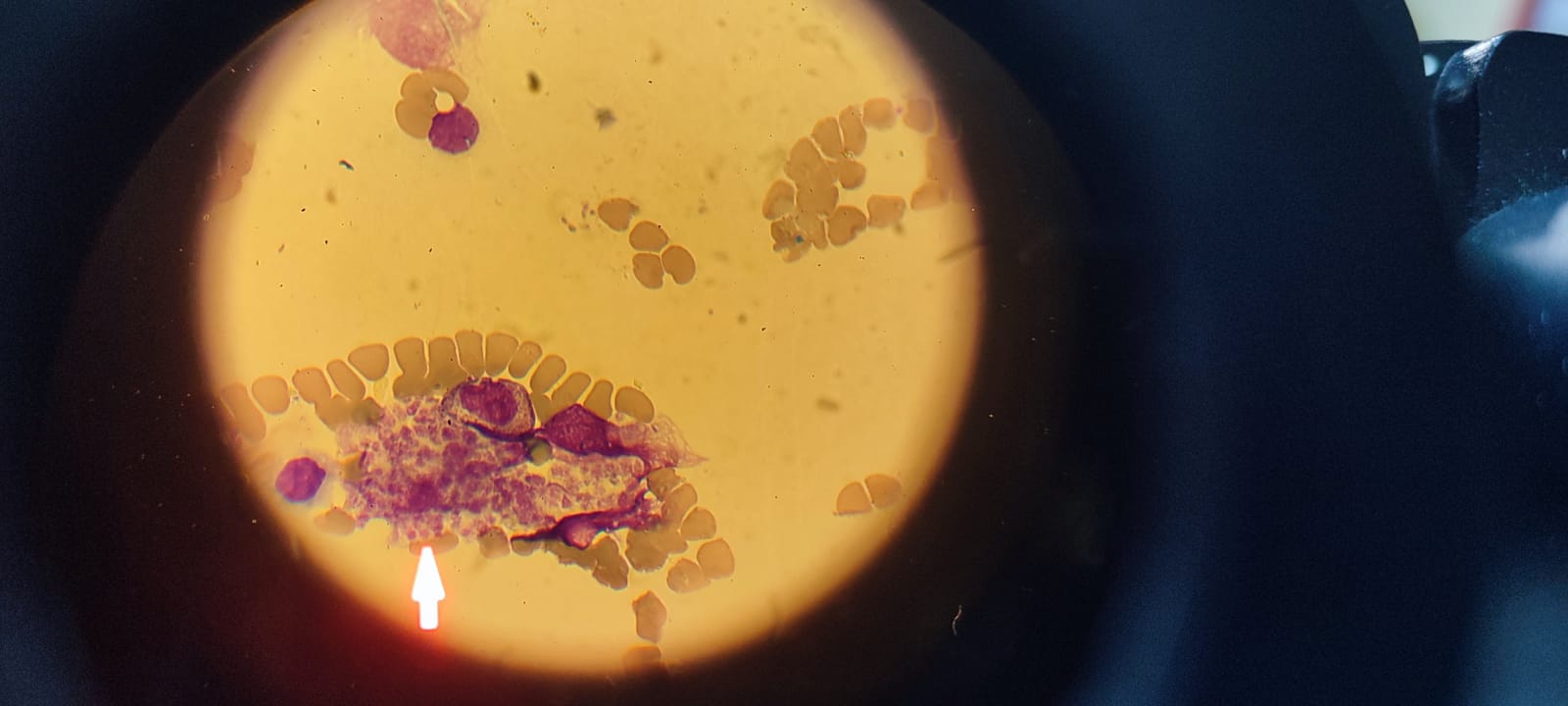

Supplement: Supplementary file 1 — Supplementary Material 1. [file 12887_2025_6451_MOESM1_ESM.jpg]
